# Room without a view—Den excavation in relation to body size in brown bears

**DOI:** 10.1002/ece3.6371

**Published:** 2020-07-20

**Authors:** Shotaro Shiratsuru, Andrea Friebe, Jon E. Swenson, Andreas Zedrosser

**Affiliations:** ^1^ Department of Biological Sciences University of Alberta Edmonton AB Canada; ^2^ Scandinavian Brown Bear Research Project Orsa Sweden; ^3^ Norwegian Institute for Nature Research Trondheim Norway; ^4^ Faculty of Environmental Sciences and Natural Resource Management Norwegian University of Life Sciences Ås Norway; ^5^ Department of Natural Sciences and Environmental Health University of South‐Eastern Norway Telemark Norway; ^6^ Department of Integrative Biology Institute of Wildlife Biology and Game Management University of Natural Resources and Life Sciences Vienna Austria

**Keywords:** brown bear, den, energy conservation, hibernation, *Ursus arctos*

## Abstract

Hibernation is an adaptive strategy to survive harsh winter conditions and food shortage. The use of well‐insulated winter dens helps animals minimize energy loss during hibernation. Brown bears (*Ursus arctos*) commonly use excavated dens for hibernation. Physical attributes of excavated dens are expected to impact the bear's heat retention and energy conservation. The objective of this study was to examine the determinants of cavity size of excavated dens and the impact of physical attributes of excavated dens on energy conservation in hibernating bears, hypothesizing that bears excavate dens in a way to minimize heat loss and optimize energy conservation during hibernation. We predicted that den cavity size would be determined by the bear's body size and that older bears would excavate better‐fitting cavities to minimize heat loss, due to their previous experience. We further predicted that physical attributes of excavated dens would affect the bears’ posthibernation body condition. Our results revealed that bears excavated a den cavity in relation to their body size, regardless of sex, and that older bears tended to excavate better‐fitting den cavities compared to young bears, as we expected. Older bears excavated better‐fitting den cavities, suggesting a potentially experience‐based shift with age in den‐excavation behavior and an optimum cavity size relative to a bear's body size. Our key finding is that insulation of excavated dens provided by wall/rood thickness and bedding materials had a significant positive effect on bears’ posthibernation body condition. We believe that our study provides new insight into how not only the quality of denning habitat, but also the quality of dens may affect hibernating animals, by presenting a potential adaptive aspect of den preparation (age effect on efficiency in den excavation) and effect of den attributes on the posthibernation body condition of brown bears.

## Graphical abstract

We found that brown bears in Sweden excavate winter dens in relation to their body size and older bears tend to excavate better‐fitting den cavities, suggesting a potentially experience‐based shift with age in den‐excavation behavior and an optimum cavity size relative to a bear's body size. Our results also revealed that insulation provided by excavated dens positively affected posthibernation body condition of bears.

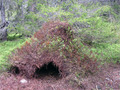

## 1 INTRODUCTION

Hibernation is a physiological and behavioral adaptation through which animals survive harsh seasonal conditions, such as inclement weather or low food availability, by minimizing energy loss (Johnson & Pelton, 1981; Pelton, Beeman, & Eagar, 1980; Thorkelson and Maxwell 1974). Mammalian hibernators usually either hibernate in natural cavities, such as hollow trees or natural caves, or construct enclosed hibernacula, such as nests, or excavated dens or burrows (Macartney, Larsen, & Gregory, 1989; Tietje & Ruff, 1980; Williams & Rausch, 1973). Many hibernators also use nesting materials (e.g., grass, moss, and branches) to increase the insulation efficiency of their hibernacula (Gedeon, Markó, Németh, Nyitrai, & Altbäcker, 2010; Michener, 1992). Small mammalian hibernators, such as arctic ground squirrels (*Spermophilus parryii*) and alpine marmots (*Marmota marmota*), decrease their body temperatures to around 0°C or even lower during hibernation to overcome their high mass‐specific metabolic rates and low amounts of body fat stores (Geiser, 2004; Ruf & Geiser, 2015). Small hibernators also have to periodically interrupt hibernation to consume food and liquids (Carey, Andrews, & Martin, 2003; Humphries, Thomas, & Kramer, 2001). In comparison, large mammalian hibernators, such as brown bears (*Ursus arctos*) and American black bears (*Ursus americanus*), decrease metabolic rates while maintaining relatively high body temperatures (Geiser, 2004; Ruf & Geiser, 2015) and rely solely on body fat stores during hibernation (Humphries, Thomas, & Kramer, 2003). Hibernacula that are well constructed and well insulated should help hibernators enhance energy conservation during hibernation (Tietje & Ruff, 1980). In addition, because ambient temperatures fluctuate even in hibernacula, using hibernacula which provide warmer microclimates helps endothermic hibernating animals minimize energy loss during hibernation (Boyles & McKechnie, 2010). Due to the relative lower decrease in body temperature of large hibernators, they may be more reliant on well‐constructed and well‐insulated hibernacula compared to small hibernators.

Brown and American black bears in several populations spend 4–6 months in winter dens (Folk, Larson, & Folk, 1976; Friebe, Swenson, & Sandegren, 2001; Johnson & Pelton, 1981) without eating or drinking, while using the fat storage gained during hyperphagia as their main energy source and conserving lean body mass via urea recycling (Atkinson & Ramsay, 1995; Barboza, Farley, & Robbins, 1997; Harlow, Lohuis, Grogan, & Beck, 2002; Hilderbrand et al., 1999; Nelson, Wahner, Jones, Ellefson, & Zollman, 1973; Tøien et al., 2011). Therefore, the use of well‐insulated dens should help bears minimize energy loss during hibernation and dens should be selected optimally in relation to energy conservation (Hayes & Pelton, 1994). Dens may also influence the amount of heat loss and vulnerability to disturbances, thereby potentially affecting the bears’ survival and reproduction (Linnell, Swenson, Andersen, & Barnes, 2000; Manchi & Swenson, 2005; Nowack, 2015; Oli, Jacobson, & Leopold, 1997). Enclosed dens, such as tree or rock cavities and excavated dens, offer protection and insulation from inclement weather (Beecham, Reynolds, & Hornocker, 1983; Oli et al., 1997; Pelton et al., 1980; Thorkelson and Maxwell 1974). As a bear can adjust an excavated den in relation to its body size, radiant heat from the soil and metabolic heat from the bear can be trapped within the den and keep the den temperature higher than the ambient temperature (Folk et al., 1976; Fornito, Lee, & Tajchman, 1982; Vroom, Herrero, & Ogilvie, 1980). Bedding materials on the ground may enhance insulation, by forming a microclimate between the bear and the soil (Craighead & Craighead, 1972; Craighead, Craighead, Varney, & Cote, 1971; Tietje & Ruff, 1980). Consequently, enclosed dens provide bears with a microenvironment where temperatures are relatively warm and stable compared to outside temperatures, thereby optimizing energy conservation (Craighead & Craighead, 1972; Tietje & Ruff, 1980). The reported tendency for female bears to select enclosed dens (Johnson & Pelton, 1981; Lentz, Marchinton, & Smith, 1983; Pelton et al., 1980) may be explained by the females’ high energy demand for birth and lactation during the denning period (Harlow et al., 2002). However, to our knowledge, there are no studies evaluating the potential effect of the physical attributes of excavated dens, such as the size of the den cavity in relation to an animal's body size, wall thickness, and bedding materials, on energy loss in hibernating animals, including bears.

Worldwide, brown bears commonly use excavated dens for hibernation (Craighead & Craighead, 1972; Linnell et al., 2000). Den cavity size, composition of the wall/roof (from now on referred to as den type), wall/roof thickness, and bedding materials have been proposed as important factors that influence heat retention and energy conservation, thereby determining the quality of an excavated den (Fornito et al., 1982; Pearson, 1975; Thorkelson and Maxwell 1974). A bear's body size has been suggested to affect den cavity size (Beecham et al., 1983; Schwartz, Miller, & Franzmann, 1987; Tietje & Ruff, 1980), but sex has not been reported to affect den cavity size (Pigeon, Côté, & Stenhouse, 2016). The volume of the air space between a bear and the cavity wall likely varies, with greater air space within the den resulting in increased convective heat loss caused by enhanced airflow (Pearson, 1975; Thorkelson and Maxwell. 1974; Tietje & Ruff, 1980). However, an optimum size of an air space warmed by the bear's radiative heat could contribute to efficient heat retention (Linnell et al., 2000; Pearson, 1975). Wall/roof thickness may be important for preserving heat within the den (Fornito et al., 1982; Lentz et al., 1983).

**FIGURE 1:**
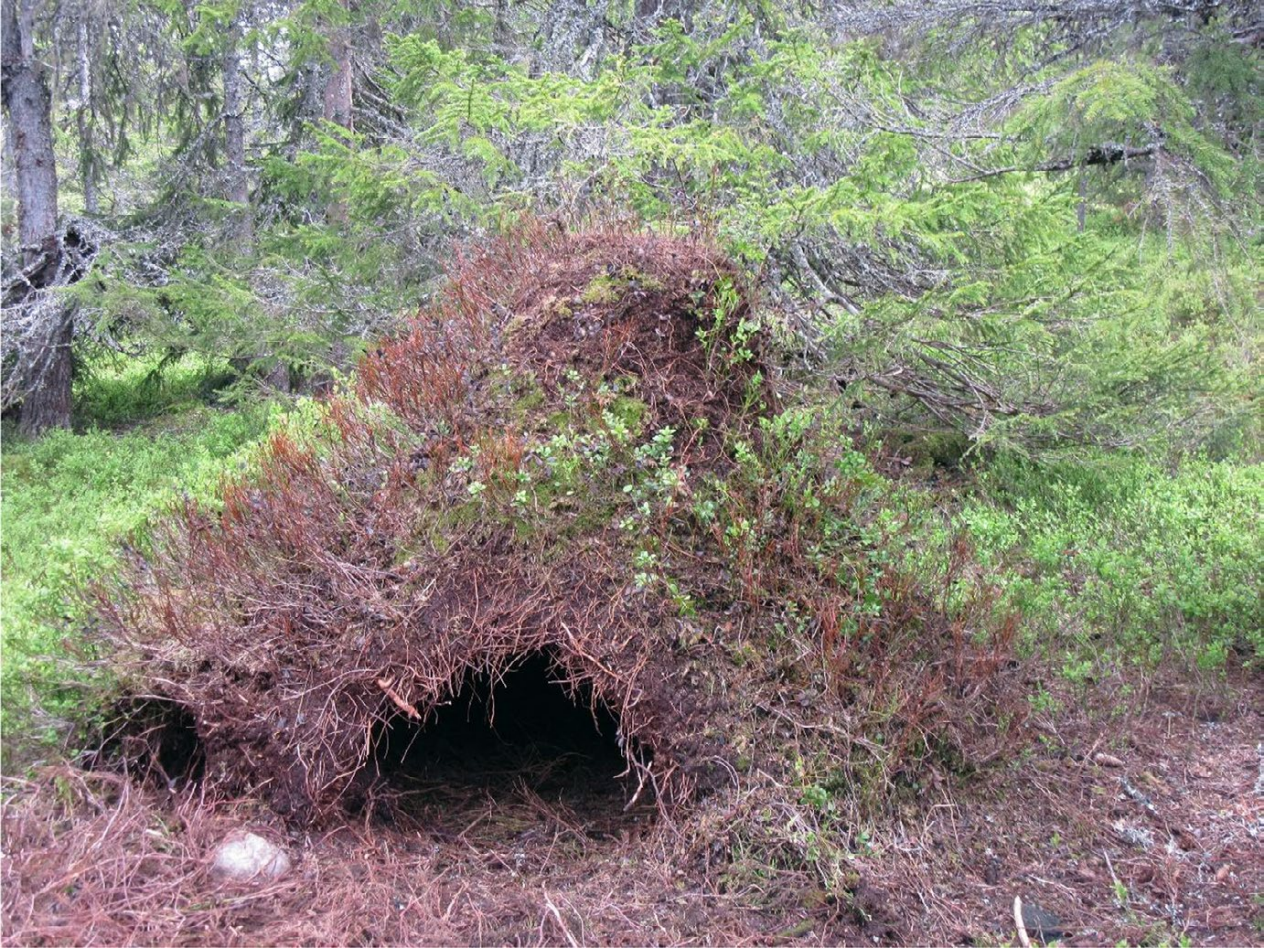
A winter den of a brown bear excavated in an abandoned anthill in Sweden

In central Scandinavia, some *Formica* ant species build very large mound‐shaped nests, and bears often excavate abandoned “anthills” that are overgrown by berry bushes and use them as winter dens (Figure 1). Anthill dens are the most common winter dens among brown bears in central Scandinavia, utilized by 56% of females and 54% of males (Manchi & Swenson, 2005). Other den types are soil dens, which are excavated in soil, rock cavities, or nest dens (Elfström & Swenson, 2009). This high use of anthill dens can be explained by the high abundance and likely also the high insulating effect of anthills, and females hibernating in anthill dens tend to have a higher reproductive success (Manchi & Swenson, 2005; Mannaart, 2016; Nowack, 2015). Therefore, these anthill dens are suspected to provide greater energy conservation compared to other den types.

In addition to the attributes of excavated dens, several other factors may affect the prehibernation body condition and energy loss of bears during hibernation. Loss of body heat during hibernation can be exacerbated by severe winter temperatures (Tøien, Blake, & Barnes, 2015), even if the animal is hibernating in an enclosed cavity (Thorkelson and Maxwell 1974). The amount of snow may positively affect energy conservation during hibernation by enhancing insulation (Beecham et al., 1983; Servheen & Klaver, 1983; Sorum et al., 2019; Vroom et al., 1980; Wathen, Johnson, & Pelton, 1986). Energy loss and body mass in bears during hibernation are highly affected by prehibernation body condition (Atkinson & Ramsay, 1995; López‐Alfaro, Robbins, Zedrosser, & Nielsen, 2013; Zedrosser, Dahle, & Swenson, 2006). Larger bears potentially gain more fat and lean mass prior to denning (Manchi & Swenson, 2005), considering the positive correlation between body size and body mass (Dahle, Zedrosser, & Swenson, 2006), and thus, males and older bears are assumed to be in a more favorable condition at the onset of hibernation, due to their larger body size (Hilderbrand et al., 1999; Swenson, Adamič, Huber, & Stokke, 2007). Energetic costs and weight loss in bears increase with the duration of hibernation (López‐Alfaro et al., 2013), and the duration of denning is sex‐dependent (Friebe et al., 2001; Manchi & Swenson, 2005; Pigeon, Stenhouse, & Côté, 2016).

Here, we examined the factors affecting attributes of excavated dens used by brown bears, testing if body size, age, and sex of bears would affect the size of the den cavity . We hypothesized that bears excavate dens in relation to their body size and that older and more experienced bears excavated dens that better fit their bodies, thus presumably being more efficient in conserving energy. We predicted that (a) cavity size would increase with body size of bears, and (b) older bears would excavate den cavities that fit their bodies better. We further examined whether attributes of excavated dens affected energy conservation, hypothesizing that bears excavate dens to minimize heat loss and optimize energy conservation during hibernation (den attribute hypothesis). We also hypothesized that life history (life history hypothesis) and environmental factors (environmental variable hypothesis) affect energy conservation of bears. These three hypotheses are not mutually exclusive, because all of the factors (den attributes, life history of bears, and environmental variables) can affect energy conservation of bears during hibernation. We predicted that (a) better‐fitting den cavities, a higher proportion of materials from anthills, and better insulation provided by thicker wall/roof and larger bedding materials would positively affect posthibernation body condition and that (b) life history (sex and age of bears) and environmental factors (autumn food availability, winter temperature, and snow deposition) would also affect posthibernation body condition.

## 2 MATERIALS AND METHODS

### 2.1 Study area

The study area was in Dalarna and Gävleborg counties in south‐central Sweden (~13,000 km^2^, ~61N, 14E). The rolling terrain is covered by an intensively managed forest, and elevation ranges from 200 m in the southeast to 1,000 m in the west. Average temperature is −7°C in January and 15°C in July, and snow cover generally lasts from late October until early May. The mean annual precipitation is 600–1,000 mm, and the vegetation period ranges from 150 to 180 days (Dahle et al., 2006). The area is mainly covered by Scots pine (*Pinus sylvestris*) and Norway spruce (*Picea abies*) interspersed with deciduous trees, such as mountain birch (*Betula pubescens*), silver birch (*Betula pendula*), aspen (*Populus tremula*), and gray alder (*Alnus incana*). Ground vegetation consists of mosses, lichens, grass, heather, and berries, including bilberries (*Vaccinium myrtillus*), lingonberries (*Vaccinium vitis‐idaea*), and crowberries (*Empetrum hermaphroditum*), which are the main foods of bears in autumn (Stenset et al., 2016).

Brown bears in Scandinavia hibernate in dens from late October to late April, although males spend less time in dens than females, and the denning duration varies in relation to age and reproductive status in females (Friebe et al., 2001; Manchi & Swenson, 2005). Reuse of dens by the same or different bears is very rare in brown bears in Sweden (eight cases out of 1,091 observations during 1986–2018; unpublished data).

### 2.2 Data collection

Bears were immobilized by darting from a helicopter in spring, shortly after den exit, and fitted with VHF (very high frequency) radio transmitters (1985–2002) or GPS (Global Positioning System)‐GSM (Global System for Mobile Communication) collars (2003–present) (Nowack, 2015; Zedrosser et al., 2006) by the Scandinavian Brown Bear Research Project (SBBRP, www.bearproject.info), according to accepted veterinary and ethical procedures. See Zedrosser et al. (2006) and Arnemo, Evans, and Fahlman (2012) for more detailed information on capture and handling. Bears were not captured before den entry, to avoid potential disturbance.

Body length (cm) was measured from the tip of the nose to the base of the tail with the tape measure overlying the dorsal midline with the bear in sternal recumbency, and chest circumference (cm) was measured at the widest part of the chest (Zedrosser et al., 2006). Body mass was measured to the nearest kg with a spring scale. Ages of bears that were not first captured as yearlings with their mothers were estimated by counting cementum annuli in an extracted premolar tooth (Matson et al., 1993). Bears captured after 5 May were excluded from the analysis, to avoid changes in weight or body condition after leaving the den, which might affect the results (Swenson et al., 2007).

The SBBRP has collected data on winter dens from 1986 to 2016. Winter dens were categorized into three types: anthill dens, anthill/soil dens (20%–80% of the den material consisted of an anthill and the rest of soil), and soil dens (>80% of the den material was soil) (Elfström & Swenson, 2009). For each den, we recorded external size (length × width × height) of the den (outside), as well as the size of the den cavity, wall/roof thickness, and size of bedding materials (length × depth). In this study, we only used data from solitary bears that used anthill, anthill/soil, and soil dens, and that did not change dens during the winter. We excluded the observations of totally or partially collapsed dens from the study.

Several bears have been captured and recorded multiple times in different years during our study. For the analyses of den cavity size and the volume of the air space between a bear and the cavity wall in relation to a bear's body size, we used the data from 86 observations of 62 solitary bears. For the analysis of posthibernation body condition index, we used the data from 57 observations of 42 solitary bears.

### 2.3 Data preparation

We used three den types in the analysis: soil dens = 1, anthill/soil dens = 2, and anthill dens = 3. External den size (from now on referred to as den size) and cavity size were estimated based on the assumption that the cavity had the shape/volume of a half‐dome. In addition, we calculated indices for the average thickness of the den wall and the size of the bed inside the den to produce an index of insulation of the den (hereafter insulation index). Equations for each variable are as follows:

$$\text{Cavity}\;\text{size}(m^{3})=\frac{4}{3}\times \pi \times \frac{\text{inner}\;\text{length}(\text{m})}{2}\times \frac{\text{inner}\;\text{width}(\text{m})}{2}\times \text{inner}\;\text{height}(\text{m})\times \frac{1}{2}$$

$$\text{Den}\;\text{size}(m^{3})=\frac{4}{3}\times \pi \times \frac{\text{outer}\;\text{length}(\text{m})}{2}\times \frac{\text{outer}\;\text{width}(\text{m})}{2}\times \text{outer}\;\text{height}(\text{m})\times \frac{1}{2}$$

Wallthickness=averageofallmeasurementsofwallandroofthickness(cm)

Bedsize=bedlength(cm)×bedthickness(cm)

Insulationindex=wallthickness×bedsize.

As an index of the volume of the air space between a bear and the cavity wall in relation to a bear's body size, we calculated the ratio of body size to cavity size (body–cavity ratio) by estimating a bear's body volume on the assumption that it resembles a cylinder. Equations used for calculating the body–cavity ratio are as follows:

$$\text{Body}‐\text{cavity}\;\text{ratio}=\frac{\text{body}\;\text{volume}(m^{3})}{\text{cavity}\;\text{size}(m^{3})}$$

where

$$\text{body}\;\text{volume}\left(m^{3}\right)=\pi r^{2}\times \frac{1}{10000}\times \text{body}\;\text{length}\left(m\right),\;\;\text{and}$$

$$r\left(\text{cm}\right)=\frac{\text{chest}\;\text{circumference(cm)}}{2\pi }.$$

Because we only used individuals that were captured after hibernation, loss of fat or body mass during hibernation could not be determined. Instead, we used a posthibernation body condition index (BCI) to evaluate the relative body condition of bears after hibernation, which can be considered as an index of energy conservation during hibernation (Cattet, Caulkett, Obbard, & Stenhouse, 2002). The BCI defines body condition as total body mass (kg) relative to body size (cm) and is calculated as the standardized residual from the linear regression of body mass (kg, log‐transformed) against linear body length (cm, log‐transformed) (Cattet et al., 2002). We confirmed that there is no correlation between the calculated BCI and linear body length (*r* = .020, *p* = .873, *df* = 63).

We included environmental and life history variables that potentially affect prehibernation body condition and energy loss during hibernation, thereby affecting the posthibernation BCI. The environmental variables were as follows: growing degree days (GDD), winter severity index (WSI), and the number of snowfall days in winter. Bears in Scandinavia rely mostly on berries, especially bilberries, for gaining fat reserves in autumn (Dahle, Sørensen, Wedul, Swenson, & Sandegren, 1998; Dahle et al., 2006; Hertel et al., 2018); therefore, berry production has an impact on the prehibernation body condition. As an index for berry production, we used GDD > 5°C (Hertel et al. 2018; Rixen et al. 2012). We included WSI defined as the number of days with temperatures <−10°C from November to April (Hertel et al. 2018) in the analysis to evaluate whether low winter temperatures affect posthibernation BCI. We used the number of days with snow on the ground in winter (hereafter annual snow days) to examine the effect of snow deposition on posthibernation BCI. The data on the environmental variables were obtained from the Swedish Meteorological and Hydrological Institute (SE‐601 76 Norrköping, Sweden) and were interpolated to a 1‐km scale to extract specific values for each location of a bear (Evans et al., 2017). Life history variables considered in the analyses were sex and age of bears.

### 2.4 Statistical analysis

We developed statistical models based on our hypotheses and carried out model selection based on Akaike's information criterion corrected for small sample size (AICc) (Bumham & Anderson, 2002), to obtain the most parsimonious models and parameter estimates. When multiple candidate models showed the same level of performance (ΔAICc < 2), we calculated model‐averaged parameter estimates by averaging the top models using the zero method (Grueber, Nakagawa, Laws, & Jamieson, 2011) to examine the overall effect size of the predictor variables.

We used generalized linear mixed‐effects models (GLMMs) with gamma distribution and a log‐link function in the analysis of cavity size, beta GLMM with logit‐link function in the analysis of body–cavity ratio, and linear mixed‐effects models (LMMs) in the analysis of posthibernation BCI. Because several bears were sampled multiple times, we added individual ID as a random intercept in all analyses. In the analysis of cavity size, we constructed candidate models with different combinations of body length and sex (Table 1). We included sex and age as predictor variables in candidate models in different combinations, while controlling for body size, in the analysis of body–cavity ratio (Table 2).

**TABLE 1:** Model selection results and parameter estimates from the most parsimonious model in an analysis of the effect of life history variables (sex, age, and body size) on the den cavity size (m^3^) of brown bears in Sweden, 1986–2016 (*n* = 86 dens of 62 solitary bears)

Models for predicting den cavity size			
Model	*k*	ΔAICc	*w*
Body length	4	0	0.693
Body length + Sex	5	2.20	0.231
Body length*Sex	6	4.40	0.077
Sex	4	49.85	0.000
~1 (null)	3	50.21	0.000

Parameter estimates from the most parsimonious model					
Variable	*β*	Confidence interval	*SE*	*t*	*p*
Body length (m)	0.83	0.64, 1.02	0.10	8.57	<.001
*R* ^2^					
Marginal	0.402				
Conditional	0.824				

Note

*k* is the number of parameters including the intercept, ΔAICc is the change in AICc from the most parsimonious model, and *w* is the Akaike model weight. All models included individual ID as a random intercept. *β* is the parameter estimate, *SE* is the standard error, 95% CI is the 95% confidence interval, *t* is the *t*‐value, and *p* is the *p*‐value. Body length was standardized by mean centering and dividing by two times its standard deviation.

**TABLE 2:** Model selection results and parameter estimates obtained from the most parsimonious model in an analysis of the factors affecting body–cavity ratio of brown bears in Sweden during 1986–2016 (*n* = 86 dens of 62 solitary bears)

Models for predicting body–cavity ratio			
Model	*k*	ΔAICc	*w*
Body length + Age	5	0	0.557
Body length + Sex +Age	6	2.26	0.180
Body length + Sex*Age	7	3.28	0.108
Body length	4	3.65	0.090
~1 (null)	3	5.53	0.035
Body length + Sex	5	5.89	0.029

Parameter estimates from the most parsimonious model					
Variable	*β*	Confidence interval	*SE*	*z*	*p*
Body length (m)	0.02	−0.33, 0.36	0.18	0.09	.93
Age	0.47	0.10, 0.83	0.19	2.50	.01
*R* ^2^					
Marginal	0.13				
Conditional	0.82				

Note

*k* is the number of parameters including the intercept, ΔAICc is the change in AICc from the most parsimonious model, and *w* is the Akaike model weight. All models included individual ID as a random intercept. *β* is the parameter estimate, *SE* is the standard error, 95% CI is the 95% confidence interval, *z* is the *z*‐value, and *p* is the *p*‐value. Body length and age were standardized by mean centering and dividing by two times their standard deviations.

To simultaneously test our hypotheses which were not mutually exclusive, we conducted a two‐step model selection approach (Pigeon, Nielsen, Stenhouse, & Côté, 2014; Sorum et al., 2019) in the analysis of posthibernation BCI. First, we constructed candidate models with different combinations of predictor variables and conducted model selection to obtain the best combination of predictor variables from the most parsimonious model for each of three hypotheses: den attributes, life history variables, and environmental variables hypotheses (Table 3). In a second step, we constructed final candidate models with different combinations of the selected predictor variables from each hypothesis and carried out model selection to obtain the final most parsimonious model and parameter estimates.

**TABLE 3:** Model selection results for different hypotheses on the factors affecting a posthibernation body condition index of brown bears in Sweden during 1986–2016 (*n* = 57 from 42 solitary bears)

	*k*	ΔAICc	*w*
Den attributes hypothesis			
Insulation	4	0	0.512
Insulation + Body–cavity ratio	5	1.44	0.249
Insulation + Den type	5	2.28	0.164
Insulation + Den type + Body–cavity ratio	6	3.94	0.071
~1 (null)	3	11.85	0.001
Body–cavity ratio	4	12.81	0.001
Den type	4	13.82	0.001
Den type + Body–cavity ratio	5	15.17	0.000
Life history hypothesis			
Age	4	0	0.481
Sex + Age	5	1.40	0.239
Sex	4	2.68	0.126
Sex*Age	6	3.58	0.080
~1 (null)	3	3.76	0.074
Environmental variables hypothesis			
Snow	4	0	0.265
~1 (null)	3	0.50	0.207
GDD	4	0.78	0.179
GDD + Snow	5	2.12	0.092
WSI + Snow	5	2.14	0.091
WSI	4	2.78	0.066
GDD + WSI	5	2.81	0.065
GDD + WSI +Snow	6	4.07	0.035

Note

*k* is the number of parameters including intercept, ΔAICc is the change in AICc from the most parsimonious model, and *w* is Akaike model weight.

All numeric predictor variables (continuous: body length, body–cavity ratio, GDD, WSI, and annual snow days; discrete: age and den type) were standardized by mean centering and dividing by two times their standard deviations. The binary variable sex (male = 0 and female = 1) was centered with a mean of zero, to control for difference in the scales and make the effect size directly comparable to each other (Gelman, 2008). The body–cavity ratio and insulation index were log‐transformed in the analysis of posthibernation BCI to achieve homogeneity of variance and normal distribution of residuals (Zuur, Ieno, & Elphick, 2010; Zuur, Ieno, Walker, Saveliev, & Smith, 2009). We examined multicollinearity of predictor variables by calculating a variance inflation factor (VIF) (Zuur et al., 2010), but no predictor variables showed VIF > 3. For linear mixed‐effects models, model validation plots showed that homogeneity of residual variance and normality of residual variance were fulfilled. The software R 3.6.1 (R Core Team, 2019) was used for all analyses. Candidate models were analyzed with the *lmerTest* package (Kuznetsova, Brockhoff, & Christensen, 2017) for gamma GLMM and LMM, and with the *glmmTMB* package for beta GLMM (Brooks et al., 2017). Model selection was conducted with the *MuMIn* package (Barton, 2012).

## 3 RESULTS

**FIGURE 2:**
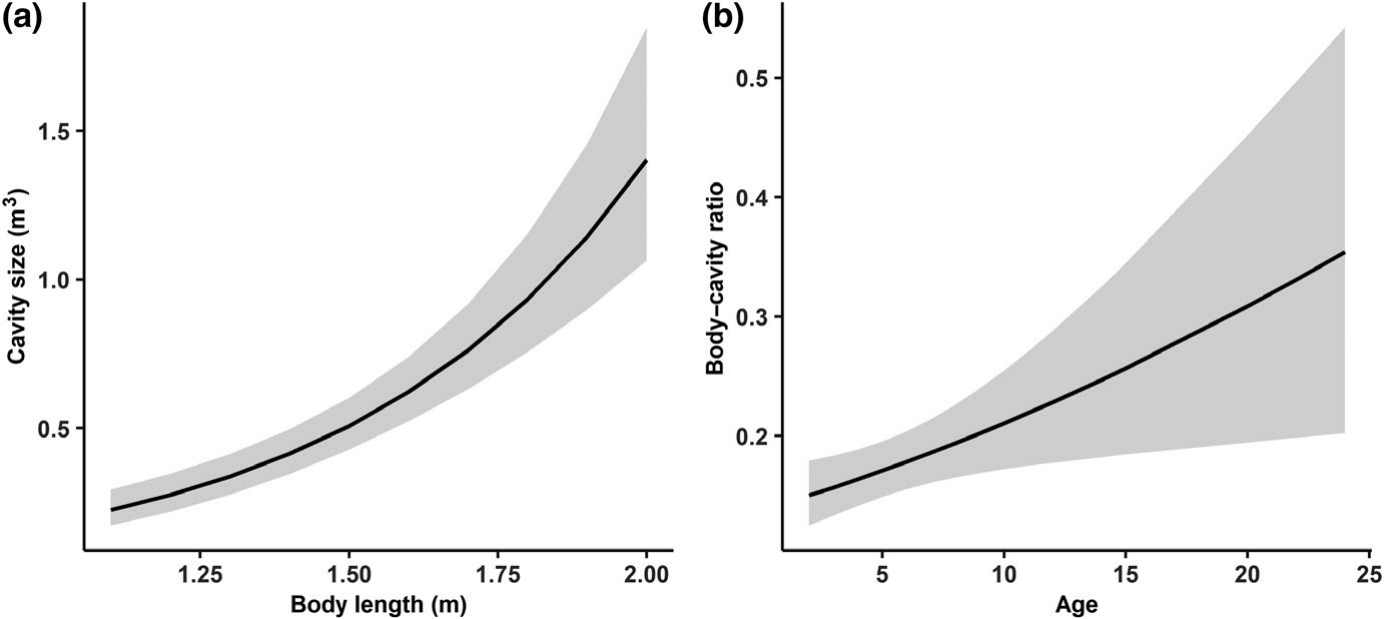
Effects of life history characteristics on physical attributes of excavated winter dens of brown bears in Sweden during 1986–2016. Predicted values by the most parsimonious models are shown with 95% confidence intervals. All the continuous predictor variables (body length and age of bears) were back‐transformed to their original means and scales. (a) Effect of body length of bears on den cavity size of an excavated dens (*n* = 86 from 62 solitary bears). (b) Effect of age of bears on the body–cavity ratio of excavated dens (*n* = 86 from 62 solitary bears) when body length of bears is controlled for

The most parsimonious model regarding the factors affecting den cavity size only included the bears’ body length as a predictor variable (Table 1), with den cavity size increasing significantly with body length (Table 1, Figure 2a). Sex of bears was not included into the most parsimonious model.

The most parsimonious model regarding the factors affecting body–cavity ratio included age and body length as predictor variables, with the body–cavity ratio increasing significantly with age when body length was controlled for (Table 2, Figure 2b).

In the first model selection step in the analysis of posthibernation BCI, insulation of dens from the den attributes hypothesis, age of bears from the life history variable hypothesis, and annual snow days from the environmental variable hypothesis were selected as the best predictor variables for the second model selection step (Table 3). In the second model selection step, the four top models showed a similar level of performance (all within ΔAICc < 2) in predicting posthibernation BCI, all of which included insulation index as a predictor variable, and the most parsimonious final model included insulation index and age as predictor variables (Table 4). Better insulation of dens positively affected posthibernation BCI (Table 4). Older bears tended to show better posthibernation body condition, but the effect was weak (Table 4). When we averaged the four top models, only insulation index had a significant effect and it showed the largest effect size (Table 4).

**TABLE 4:** Results of the second model selection step and parameter estimates obtained from the most parsimonious model and model averaging of the 4 top models (ΔAICc < 2) in an analysis of the posthibernation body condition index of brown bears in Sweden during 1986–2016 (*n* = 57 from 42 solitary bears).

Final models for predicting body–cavity ratio			
Model	*k*	ΔAICc	*w*
Insulation + Age	5	0	0.324
Insulation	4	0.36	0.271
Insulation + Snow	5	0.95	0.201
Insulation + Age + Snow	6	1.02	0.194
Age	4	8.45	0.005
Age + Snow	5	8.59	0.004
Snow	4	11.71	0.001
~1 (null)	3	12.21	0.001

Parameter estimates and *R* ^2^ values from the most parsimonious model					
Variable	*β*	Confidence interval	*SE*	*t*	*p*
Insulation	0.83	0.35, 1.31	0.25	3.37	.0014
Age	0.40	−0.07, 0.89	0.25	1.64	.11
*R* ^2^					
Marginal	0.254				
Conditional	0.254				

Parameter estimates from the averaged models					
Variable	*β*	Confidence interval	Adjusted *SE*	*z*	*p*
Insulation	0.89	0.39, 1.39	0.26	3.49	<.001
Age	0.16	−0.10, 0.90	0.25	0.63	.53
Snow	−0.09	−0.79, 0.18	0.19	0.46	.65

Note

Insulation is an insulation index (log‐transformed), and Snow is the number of annual snow days. *k* is the number of parameters including intercept, ΔAICc is the change in AICc from the most parsimonious model, and *w* is Akaike model weight. All the models included individual ID as a random intercept. *β* is the parameter estimate, *SE* is the standard error, confidence interval is 95% confidence interval, *t* is the *t*‐value, *z* is the *z*‐value, and *p* is the *p*‐value. Insulation index , age, and the number of annual snow days were standardized by mean centering and dividing by two times their standard deviations.

## 4 DISCUSSION

This is the first study of determinants of den cavity size and potential effects of den attributes on posthibernation body condition of hibernating animals, to our knowledge. We found that the cavity size of dens excavated by brown bears was determined by a bears’ body length, independently of sex, and that older bears excavated better‐fitting dens that are likely more energy‐efficient. We also found evidence that the level of insulation of excavated dens affected the posthibernation BCI.

Den cavity size was positively related to a bear's body size, as suggested, but not documented, by previous studies (Beecham et al., 1983; Schwartz et al., 1987; Tietje & Ruff, 1980). We also found that neither cavity size nor the body–cavity ratio was affected by sex. This is consistent with the results of the study by Pigeon, Côté, et al. (2016), which reported that the cavity size of an excavated den used by brown bears was not different between males and females. The body–cavity ratio increased with age when body size was controlled for, which implies that older bears excavated better‐fitting den cavities.

A potential explanation for this age effect is that older bears may be more experienced and skilled, and therefore able to excavate cavities that fit their bodies better to reduce heat loss during hibernation, compared to younger and less experienced bears. Age and experience likely are important factors that affect animal behavior and fitness (Reiter, Panken, & Le Boeuf, 1981; Sand, Wikenros, Wabakken, & Liberg, 2006; Sydeman, Huber, Emslie, Ribic, & Nur, 1991). For example, previous studies have revealed that older and experienced individuals tend to have higher reproductive success in northern elephant seals (*Mirounga angustirostris*) (Reiter et al., 1981; Sydeman et al., 1991) and reindeer (*Rangifer tarandus*) (Weladji et al., 2008). Cheetahs (*Acinonyx jubatus*) (Eaton, 1970), wolves (*Canis lupus*) (Sand et al., 2006), and polar bears (*Ursus maritimus*) (Stirling & Latour, 1978) have been also reported to have higher hunting success with increasing age and experience.

We found that den attributes potentially affect energy conservation in hibernating brown bears. Our results showed that wall/roof thickness and bed size positively affected the posthibernation BCI, partially supporting the den attributes hypothesis. These results are consistent with previous studies that suggested that thicker walls and bedding materials on the ground would enhance insulation and heat retention for denning animals (Craighead et al., 1971; Craighead & Craighead, 1972; Fornito et al., 1982; Lentz et al., 1983; Linnell et al., 2000; Pearson, 1975; Thorkelson et al. 1974; Tietje & Ruff, 1980). Boyles and McKechnie (2010) proposed that, considering that ambient temperature can vary even in hibernacula, it would be beneficial for endothermic hibernating animals to use hibernacula that provide them with warmer microclimates to minimize energy loss. Our results suggest that better insulation of excavated dens, provided by thicker walls/roofs, potentially facilitates more efficient heat retention and energy conservation of hibernating bears. The air space within the den also has been suggested to be an important determinant of insulation (Fornito et al., 1982; Lentz et al., 1983; Linnell et al., 2000; Pearson, 1975), but we did not find an effect of den cavity ratio on posthibernation BCI. We also expected that bears hibernating in excavated dens with a higher proportion of anthill materials would have a higher posthibernation BCI, based on high use of anthill dens by bears and higher reproductive success of females that hibernated in anthill dens (Manchi & Swenson, 2005; Mannaart, 2016; Nowack, 2015). However, den type did not affect posthibernation BCI. In addition to wall/roof thickness and bed size, a bear's age was found to have a weak effect on posthibernation BCI, partly supporting the life history hypothesis. We expected that males and older bears would show higher posthibernation BCI (life history hypothesis), because larger bears can gain more fat and lean mass before hibernation (Dahle et al., 2006; Hilderbrand et al., 1999; Manchi & Swenson, 2005; Swenson et al., 2007) and the females’ longer hibernation duration may result in increased energy and weight loss (López‐Alfaro et al., 2013; Manchi & Swenson, 2005). However, our results suggest that neither sex nor age of bears had a significant effect on posthibernation BCI, although our data did not include females that had given birth and lactated during hibernation. We also found no effect of environmental conditions on posthibernation BCI, that is, no support for the environmental variables hypothesis. Berry production (Dahle et al., 1998, 2006; Hertel et al., 2018) was expected to affect prehibernation body condition, and winter temperatures (Thorkelson et al. 1974; Tøien et al., 2015) and snow deposition (Beecham et al., 1983; Servheen and Klaver 1983; Vroom et al., 1980; Wathen et al., 1986) were expected to affect energy loss during hibernation (Atkinson & Ramsay, 1995; López‐Alfaro et al., 2013; Zedrosser et al., 2006), thereby affecting posthibernation body condition. However, neither GDD, WSI, nor the number of snowfall days were found to be important predictors of posthibernation BCI. We used GDD as an index of production of bilberries, which are the major food source of bears in autumn (Dahle et al., 1998, 2006; Hertel et al., 2018), expecting that higher GDD would positively affect prehibernation body condition of bears. However, GDD was not selected as an important predictor of posthibernation BCI, suggesting that the effect of berry production on prehibernation body condition or the effect of prehibernation body condition on posthibernation body condition is probably negligible, compared to the effect of insulation provided by an excavated den, on energy conservation during hibernation. Cold winter temperatures did not negatively affect posthibernation BCI, probably because excavated dens protected bears from the extra energy loss caused by lower temperatures (Beecham et al., 1983; Craighead & Craighead, 1972; Oli et al., 1997; Pelton et al., 1980; Thorkelson & Maxwell, 1974). Lack of an effect of snow on posthibernation BCI can be partially explained by the fact that the importance of snow insulation likely is negligible in regions where winter temperatures rarely drop below −20°C (Elfstrom et al. 2008; Schoen et al. 1987).

We acknowledge that the lack of data on prehibernation body condition made it difficult to evaluate the effect of den attributes on posthibernation body condition. In addition, additional potentially confounding variables that we were not able to evaluate might affect prehibernation body condition and energy conservation during hibernation, such as quality of the den site in terms of topography and insulation effects from the surrounding habitat (Sorum et al., 2019). However, our result indicates a positive effect of insulation of excavated dens on posthibernation BCI, which suggests that physical attributes of hibernacula potentially affect energy conservation of hibernating mammals. Although selection of denning habitat by animals has been thoroughly studied (Burger et al., 1988; Prior & Weatherhead, 1996; Smereka et al., 2017; Zukal, Berková, & Řehák, 2005), the effects of hibernaculum attributes and preparation (i.e., bedding materials) on energy conservation during hibernation and posthibernation body condition seem to be a knowledge gap in the study of hibernating animals. Our study has taken a first step to fill this gap and provides new insight into how the quality of hibernacula may affect hibernating animals.

## CONFLICT OF INTEREST

None declared.

## AUTHOR CONTRIBUTION

**Shotaro Shiratsuru:** Conceptualization (lead); Formal analysis (lead); Methodology (lead); Writing‐original draft (lead). **Andrea Friebe:** Data curation (lead); Investigation (equal); Validation (supporting); Writing‐review & editing (supporting). **Jon E. Swenson:** Funding acquisition (lead); Investigation (lead); Project administration (lead); Supervision (equal); Validation (lead); Writing‐review & editing (lead). **Andreas Zedrosser:** Funding acquisition (lead); Investigation (lead); Project administration (lead); Supervision (lead); Validation (lead); Writing‐review & editing (lead).

## Data Availability

All the data used in the analysis and model validation plots are openly available in figshare (https://usn.figshare.com/), the data repository of the University of South‐Eastern Norway: https://doi.org/10.23642/usn.12174702.
